# Zika Virus Replication in Dorsal Root Ganglia Explants from Interferon Receptor1 Knockout Mice Causes Myelin Degeneration

**DOI:** 10.1038/s41598-018-28257-5

**Published:** 2018-07-05

**Authors:** Vera Giulia Volpi, Isabel Pagani, Silvia Ghezzi, Matteo Iannacone, Maurizio D’Antonio, Elisa Vicenzi

**Affiliations:** 1 Myelin Biology Unit, Division of Genetics and Cell Biology, San Raffaele Scientific Institute, Milan, Italy; 2 Viral Pathogens and Biosafety Unit, Division of Immunology, Transplantation and Infectious Diseases, San Raffaele Scientific Institute, Milan, Italy; 3 Dynamics of Immune Responses Unit, Division of Immunology, Transplantation and Infectious Diseases, San Raffaele Scientific Institute, Milan, Italy

## Abstract

Zika virus (ZIKV) is a neurotropic agent that targets the developing fetal brain in women infected during pregnancy. In addition to the developing central nervous system, ZIKV has been recently shown to infect cells of the peripheral nervous system (PNS), highlighting its potential to cause acute peripheral neuropathies in adults, such as Guillain-Barré Syndrome (GBS). Here we show that myelinating dorsal root ganglia (DRG) explants obtained from interferon-alpha/beta receptor knock-out mice are productively infected by ZIKV. Virus replication is cytopathic in both peripheral neurons and myelinating Schwann cells leading to myelin disruption. These results confirm and extend previous observations suggesting that the PNS is indeed a potential site of ZIKV infection, replication and cytopathicity.

## Introduction

Zika Virus (ZIKV) is a member of the *Flaviviridae* family transmitted to humans mainly by bites of the *Aedes* mosquito species^1^. The virus was firstly isolated from the blood of a febrile monkey in 1947 in the Zika forest of Uganda^2,3^. However, it has been unrecognized as a dangerous human pathogen until 2013–2014, when an unusual outbreak of ZIKV-related Guillain-Barré syndrome (GBS), a severe condition characterized by inflammatory demyelination of the peripheral nervous system (PNS), was observed in French Polynesia^4^. In late 2013, the virus was introduced in Brazil^5^, but the severity of the infection appeared only at the end of 2015, when the first cases of ZIKV-related microcephaly in newborns were reported^6^. It is now well established that ZIKV can cause fetal brain damage particularly when the mother is infected during the first trimester of pregnancy^6^. In addition, cases of GBS-like inflammatory demyelinating disorders have also been linked to ZIKV infection in different countries of South and Central America^7,8^. A recent meta-analysis of 36 studies addressing questions related to ZIKV infection and GBS strongly pointed at ZIKV as a cause of GBS^9^.

GBS is an autoimmune neuropathy in which the immune-mediated demyelination of peripheral nerves leads to motor defects, muscle weakness, flaccid paralysis, sensory disturbance and, in the most severe cases, even death^10^. Both primary demyelinating (with secondary axonal degeneration) and pure axonal forms of GBS have been described, the first being more diffuse in Europe and America and the second more common in Asia^10^. A recent study, indeed, indicated that an acute inflammatory demyelinating polyneuropathy (AIDP), rather than the acute motor axonal neuropathy (AMAN), was the predominant form of GBS in Colombia^8^. A direct action of ZIKV replication on peripheral nerves degeneration was suggested by the fact that a shorter 6–7 day interval (vs. 2–3 weeks of classical GBS) has been reported to occur between the acute ZIKV-related symptoms and the appearance of neuropathological features^8,11^. The persistent detection of viral RNA in the PNS of infected rhesus macaques strongly supports this hypothesis^12^. In addition, unlike patients affected either by the axonal forms or by the demyelinating GBS triggered by pathogens other than ZIKV, individuals with presumable ZIKV-associated GBS display low levels of anti-ganglioside antibodies (Abs)^4^. This implies that ZIKV may cause GBS by a still unidentified pathogenic mechanism that may differ from other etiologies^4^.

Although ZIKV tropism for the central nervous system (CNS) has been extensively studied in several model systems^13–16^, its capacity to infect the PNS has been poorly explored. A recent study performed in interferon-alpha/beta receptor subunit 1 knock-out (*Ifnar1*-KO) mice, permissive to ZIKV replication, suggested that peripheral neurons and Schwann cells (SC) derived from dorsal root ganglia (DRG) explants are less susceptible to ZIKV infection as compared to CNS cells^17^. However, another study showed that ZIKV can directly infect SC and peripheral neurons derived from re-programming of human pluripotent stem cells^18^. The same study also showed that ZIKV infected the somatosensory neurons of A129 mice, defective of IFNAR1 expression, causing neuronal cell death^18^. However, whether ZIKV can directly infect mature SC, which have already formed myelin, and cause demyelination is unknown. For this reason, we tested whether ZIKV productively infected myelinating SC and, eventually, altered myelin stability in myelinating DRG explants from *Ifnar1*-KO mice. Here we show that ZIKV establishes a productive and cytopathic infection in both DRG neurons and myelinating SC leading to myelin breakdown, endoplasmic reticulum (ER) stress and cell death. Thus, our study supports the hypothesis of a direct role of ZIKV replication in causing PNS pathology that may eventually lead to GBS.

## Results

### Zika virus infection of PNS neurons and SC in myelinating DRG explants

**Figure 1:**
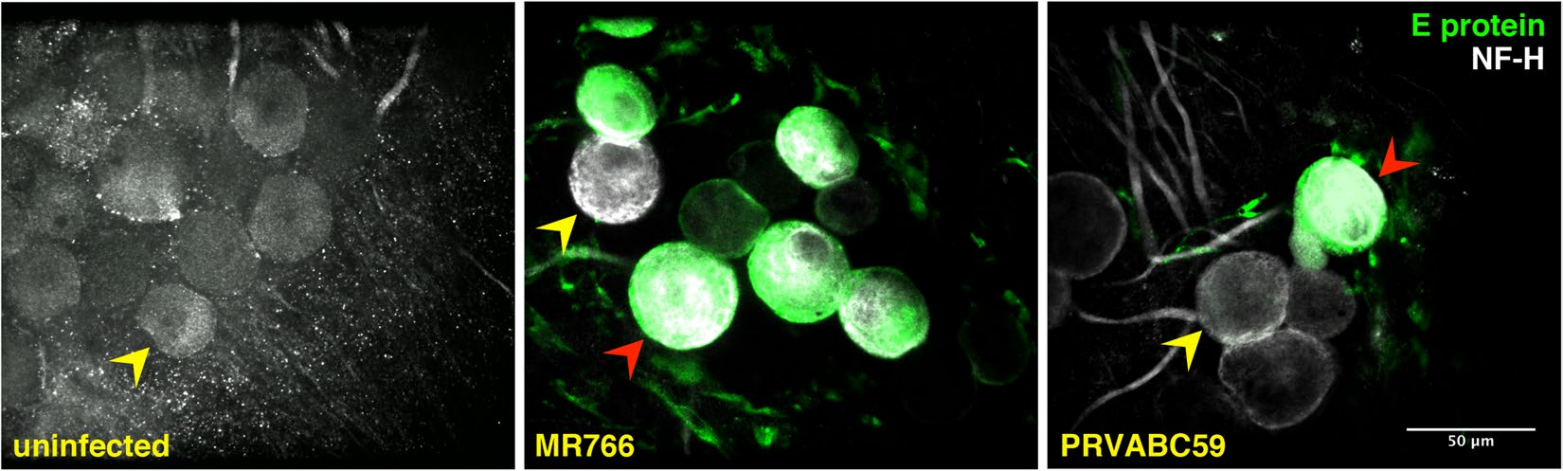
ZIKV infects peripheral DRG neurons in myelinating DRG explants cultures. Immunofluorescence staining on myelinating *Ifnar1*-KO DRG explants infected with MR766 or PRVABC59 strains after 6 dpi. In green, the anti-E protein mAb marks the viral particles detected in the cell body of the infected neurons and undetectable in uninfected DRG explants; in gray, anti-neurofilament-H (NF-H) Ab stains both neuronal bodies and axons. Red and yellow arrowheads indicate examples of infected and uninfected neurons, respectively. Scale bar, 50 μm.

In order to investigate whether ZIKV could induce pathology in the PNS, we took advantage of myelinating DRG explants obtained from *Ifnar1*-KO mice, previously shown to be highly permissive to ZIKV infection and replication, in contrast to immunocompetent mice that are relatively resistant^19^. To this aim, DRG neurons-Schwann cells co-cultures were established from *Ifnar1*-KO embryos at embryonic day 13.5 (E13.5) and cultured for 14 days to undergo myelination. Then, the DRG explants were infected with two different ZIKV strains: the historical MR766^2^ and the 2015 Puerto Rican PRVABC59^20^. The kinetics of ZIKV replication were determined by immunofluorescence at 1, 3, 6 and 10 days post-infection (dpi). Peripheral DRG neurons were visualized by neurofilament-H (NF-H) staining, whereas myelin protein zero (P0) was used to identify myelinating SC and ZIKV infection was revealed by anti-viral envelope (E) protein monoclonal Ab (mAb). In accordance with a previous study^17^, ZIKV was detected in the neuronal bodies of neurons infected with both MR766 and PRVABC59 strains (Fig. 1, middle and right panels), whereas the uninfected peripheral neurons were all negative for the virus specific anti-E protein (Fig. 1, left panel). As expected from other *in vitro* infection models^21^, the infection by the historical MR766 strain was more efficient than that of the contemporary PRVABC59 strain, as discussed further.

**Figure 2:**
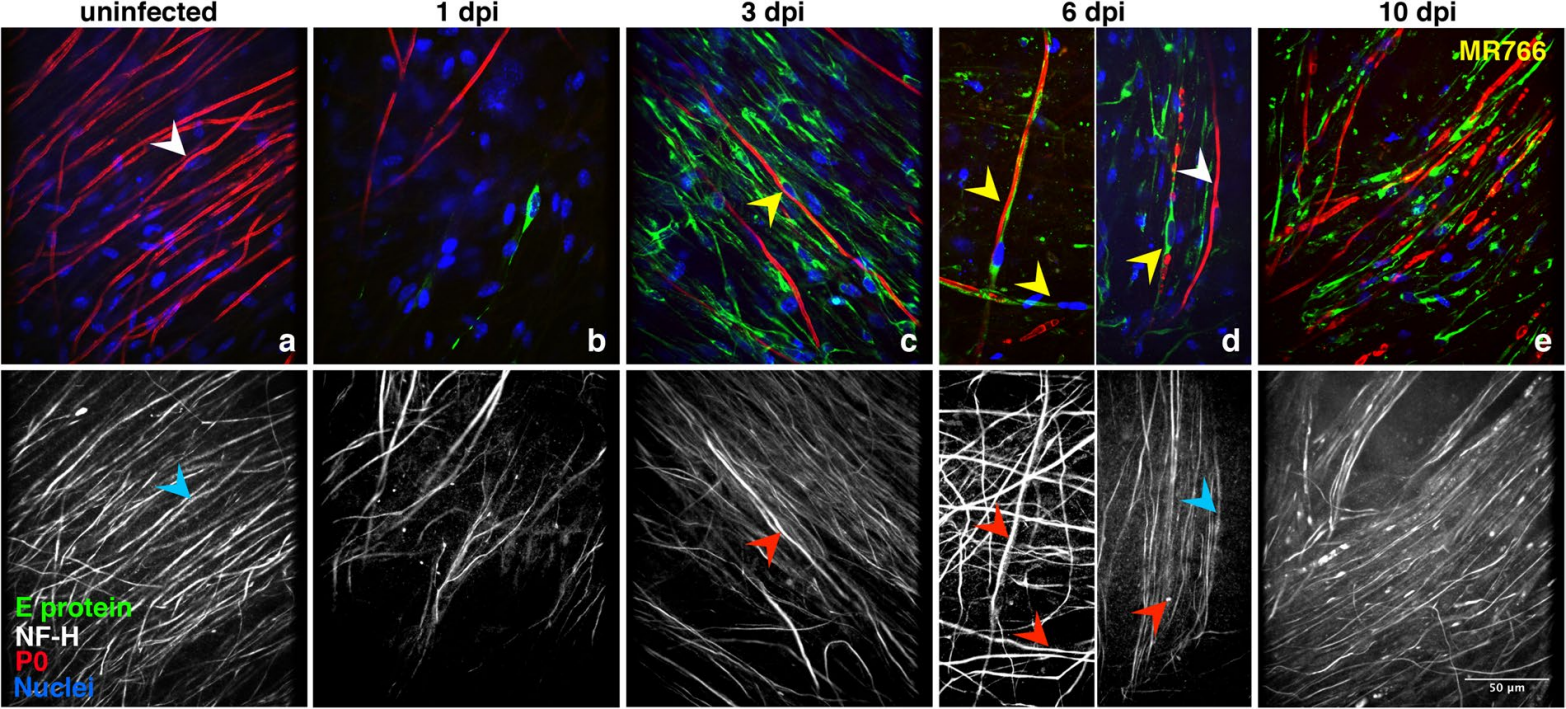
Kinetics of MR766 replication in myelinating DRG explants. Myelinating *Ifnar1*-KO DRG explants were infected with MR766 ZIKV strain after 2 weeks of myelination induction. Staining against viral E protein (green), NF-H (white) and P0 (red) was performed 1 (**b**), 3 (**c**), 6 (**d**) and 10 (**e**) days post-infection (dpi) and compared to uninfected control (**a**). Hoechst dye was used to stain the nuclei (blue). White and blue arrowheads indicate single uninfected SC with intact myelin and the associated axon, respectively (panels a and d). Yellow and red arrowheads point at infected myelinating SC and their corresponding axons. Some SC display ongoing demyelination with or without axonal degeneration. At 10 dpi, diffuse demyelination, axonal degeneration and abundant cytolysis are detectable. Scale bar, 50 μm.

**Figure 3:**
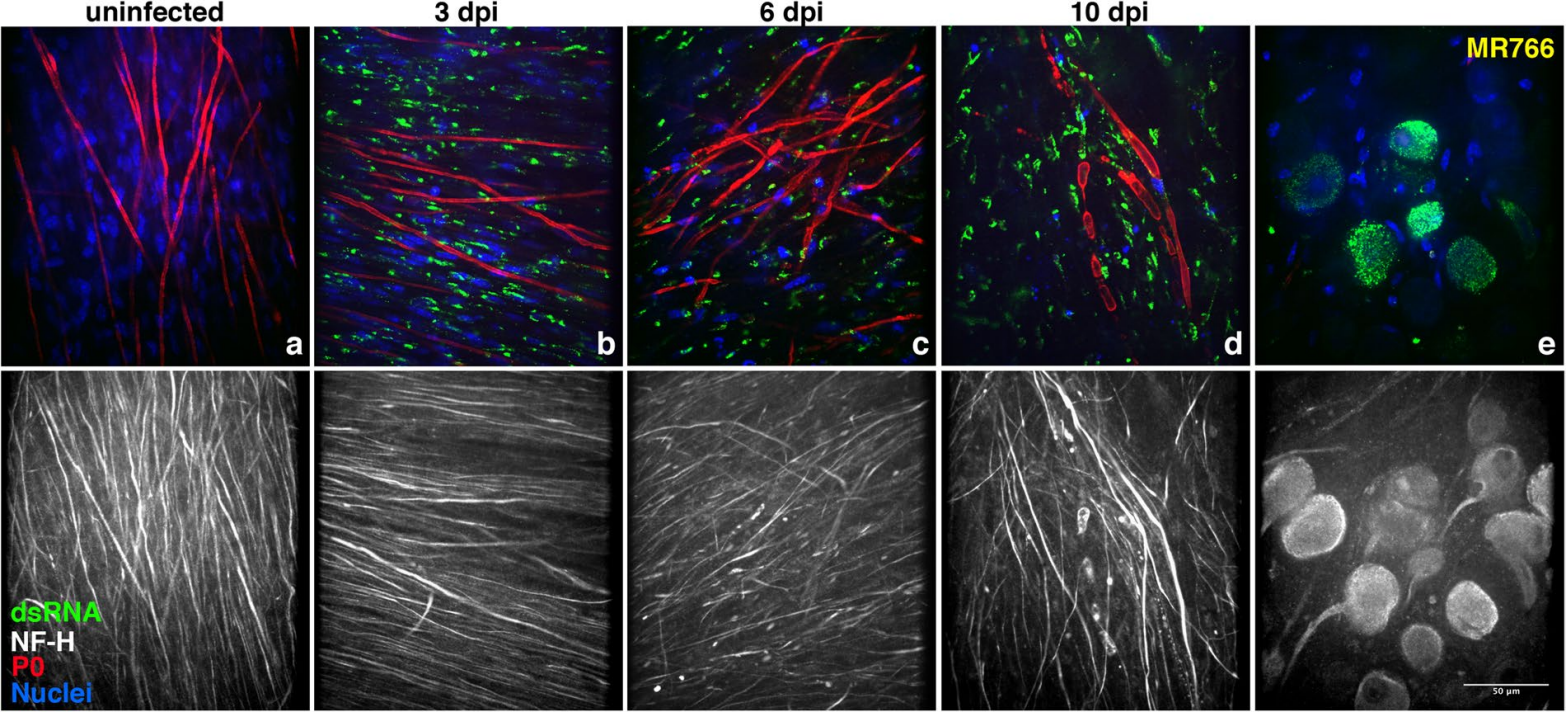
Active replication of MR766 ZIKV strain in myelinating DRG explants. Immunofluorescence staining of myelinating *Ifnar1*-KO DRG explants in presence or absence of ZIKV infection. Viral dsRNA staining (in green) is undetectable in uninfected DRG explants (**a**) and positive in infected myelinating co-cultures after 3 (**b**), 6 (**c**) and 10 (**d**) days post-infection (dpi). Panel **e** illustrates active viral replication in infected neuronal bodies. Scale bar, 50 μm.

Since the SC present in the DRG explants can sort the axons and form myelin around them, we analyzed whether ZIKV replication was also occurring in these cells of glial origin. Figure 2 illustrates the kinetics of MR766 infection. As compared to uninfected cultures (Fig. 2a), after 1 dpi, only very few cells were positive for the viral E protein expression. These cells were negative for P0 staining and were mainly located at the periphery of the DRG explants (Fig. 2b). Extensive MR766 staining  was instead detected 3 dpi in almost all cell types present in the culture, including P0-positive myelinating SC. At this stage, the cytopathic effect of ZIKV infection, including cell death and cytolysis, was not or barely observed (Fig. 2c). Of note, after 6 dpi, many infected myelinating SC displayed highly fragmented myelin, a typical sign of demyelination. Some of these cells appeared still associated to intact axons, whereas other axons were concomitantly degenerating (Fig. 2d). After this time point, important features of cytopathology and diffuse cytolysis were progressively observed, such as presence of cellular debris and fragmented nuclei. At 10 dpi, both axonal degeneration and, in particular, myelin breakdown were more pronounced than at earlier time points (Fig. 2e). Similar results were obtained by staining infected cells with a mAb that recognizes the ZIKV double-stranded RNAs (dsRNA) generated by the viral RNA polymerase (Fig. 3).

**Figure 4:**
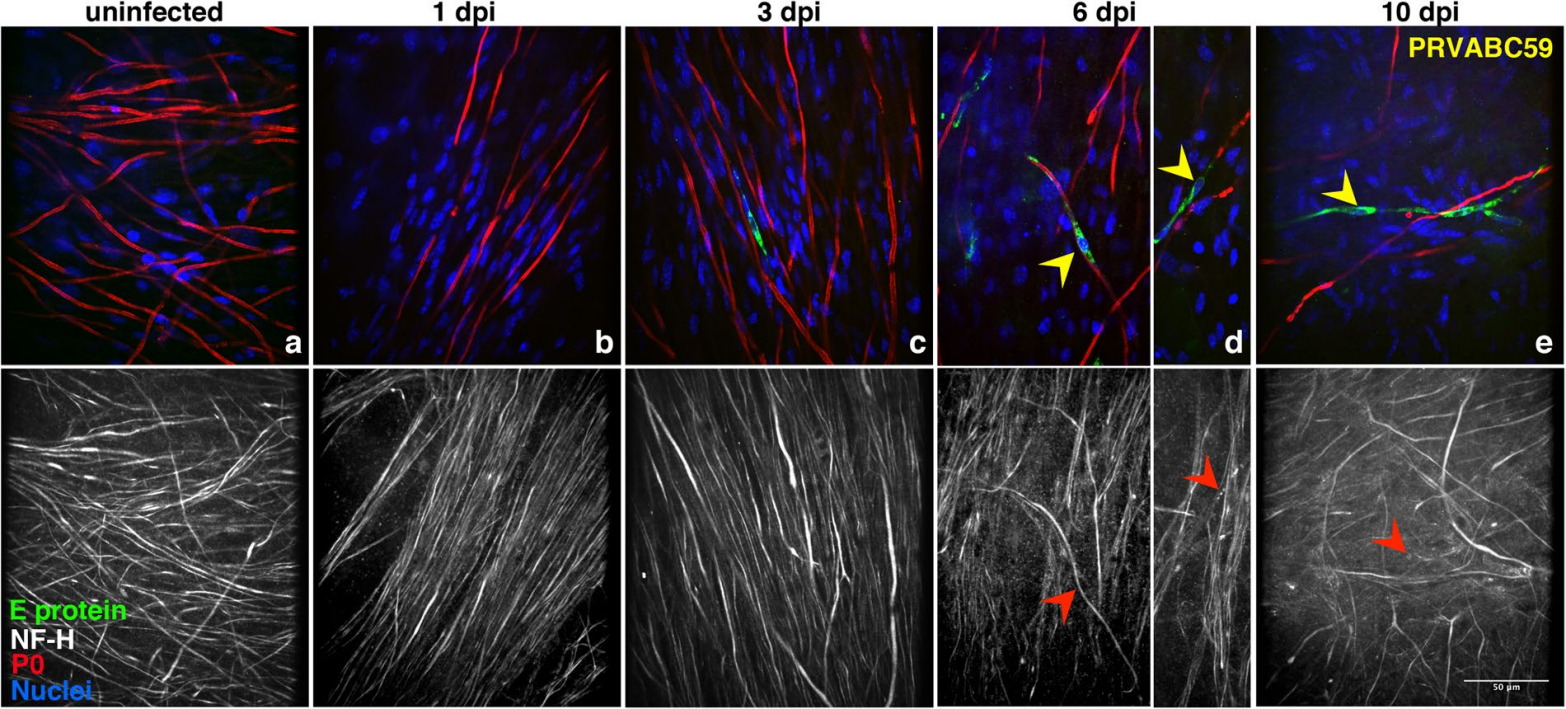
Kinetics of PRVABC59 replication in myelinating DRG explants. Myelinating *Ifnar1*-KO DRG explants were infected with PRVABC59 strain after 2 weeks of myelination induction. Staining with anti-E viral protein (green), anti-NF-H (white) and anti-P0 (red) Abs was performed 1 (**b**), 3 (**c**), 6 (**d**) and 10 (**e**) days post-infection (dpi) and compared to uninfected control (**a**). Hoechst dye was used to stain the nuclei (blue). Yellow and red arrowheads point at infected myelinating SC and at the corresponding axons, respectively. Some infected myelinating SC display demyelination with or without associated axonal degeneration. Scale bar, 50 μm.

In parallel, we also evaluated the effects of the PRVABC59 strain, visualized with the E protein staining (Fig. 4**)**. The ability of PRVABC59 to infect cells of the DRG explants appeared clearly lower as compared to that of MR766 strain. Indeed, starting from 3 dpi, only a few cells were clearly infected, among which peripheral neurons and myelinating SC. PRVABC59 infection caused features of both demyelination and neuronal degeneration, although at lower levels than those observed after infection with the MR766 strain (compare with Fig. 2).

Altogether these data clearly demonstrate that both myelinating SC and peripheral neurons are target of ZIKV infection and replication leading to both myelin destruction and axonal degeneration.

### ZIKV infection activates apoptosis in myelinating DRG explants

**Figure 5:**
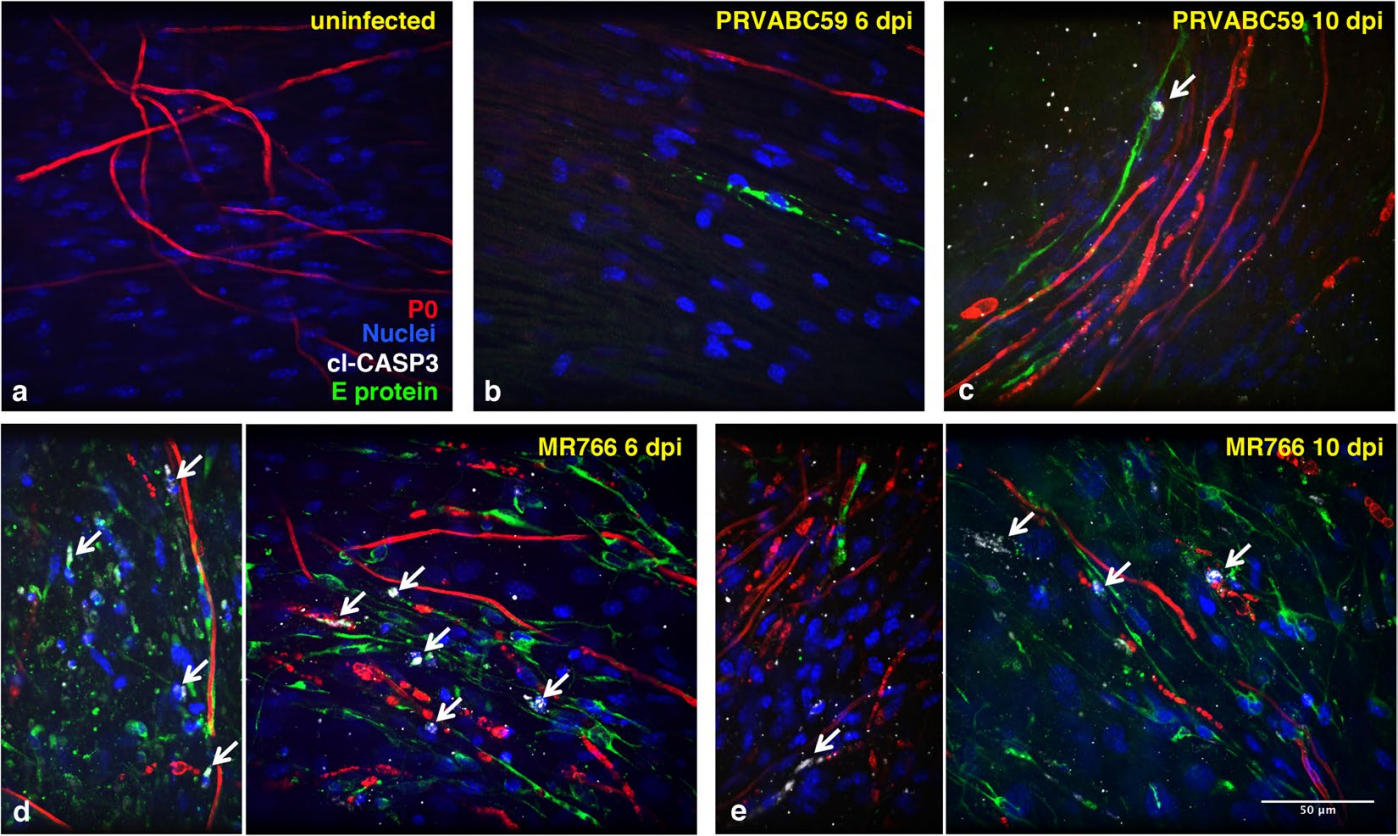
ZIKV infection activates caspase 3 in myelinating DRG explants. Myelinating *Ifnar1*-KO DRG explants were infected with either PRVABC59 (**b**,**c**) or MR766 (**d**,**e**) strains after 2 weeks of myelination induction. In panels (**d**,**e**), two representative images of MR766 infection at 6 and 10 days post-infection (dpi), respectively, are shown. Staining with anti-E viral protein (green), anti-cl-CASP3 (white) and anti-P0 (red) Abs was performed and compared to uninfected control (**a**). Hoechst dye was used to stain the nuclei (blue). White arrows point at cells in which activated cl-CASP3 signal, marker of apoptosis induction, is detected. Scale bar, 50 μm.

As ZIKV infection was shown to induce apoptosis of somatosensory neurons in mouse PNS^18^, we monitored the presence of apoptotic cells in the infected cultures by staining the DRG explants with the anti-cleaved-caspase 3 (cl-CASP3) mAb. Signal of activated cl-CASP3 was only rarely or not detectable in the uninfected controls (Fig. 5a), whereas in cultures infected with the MR766 strain, several cells, including P0-positive myelinating SC, displayed cl-CASP3 staining at 6–10 dpi (Fig. 5d,e, respectively**)**; of note, cl-CASP3 staining co-localized with the viral E protein. Finally, a mild cl-CASP3 signal was also detected upon infection with the PRVABC59 strain (Fig. 5b,c), although only at 10 dpi (Fig. 5c). Thus, ZIKV can activate pro-apoptotic pathways in cells of the PNS.

### Productive ZIKV infection and cell death of myelinating DRG explants

**Figure 6:**
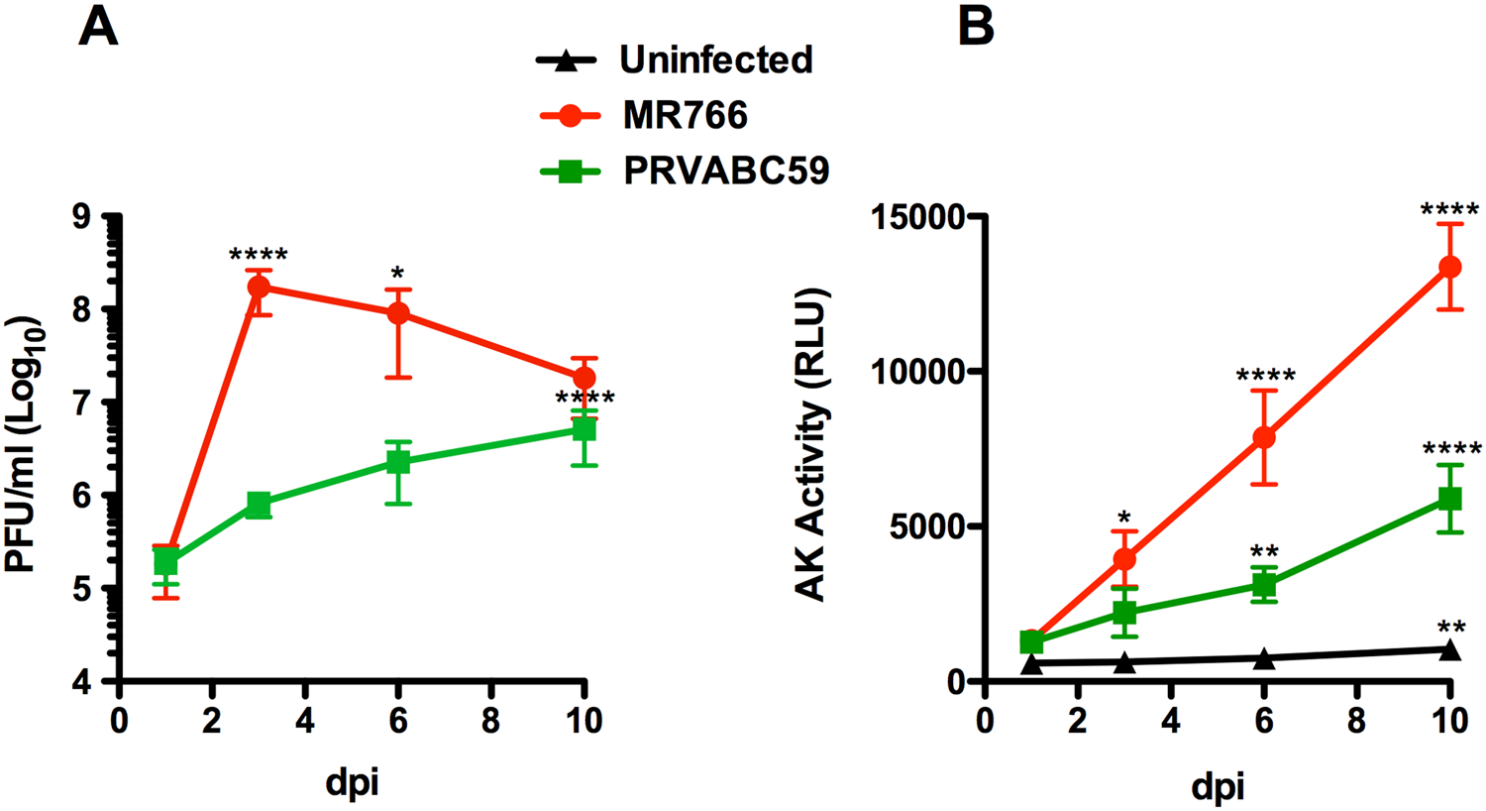
Kinetics of ZIKV productive infection and virus-induced cell death in DRG explants. Co-cultures were infected as described above. Culture supernatants were harvested at regular intervals after infection up to 10 days post-infection (dpi) and viral titers were determined by plaque assay on Vero cells (**a**). Evaluation of necrotic cell death was determined by the levels of AK activity released in the culture supernatants and expressed as relative luminescence units (RLU) (**b**). Means ± SD of 7 independent wells are reported. P values were determined using one-way ANOVA with Bonferroni***’***s multiple comparison test of day 1 post-infection vs. each following day (*p < 0.05; **p < 0.01; ****p < 0.0001).

In order to confirm the productive infection of cells present in the DRG explants, the titer of infectious virus released in the culture supernatant was determined by plaque assay on Vero cells. By testing 7 independent co-cultures per condition, we observed productive infection with both viral strains (Fig. 6a). The kinetic of MR766 replication, however, was faster than that of the PRVABC59 strain and, overall, MR766 isolate replicated more efficiently than PRVABC59, confirming what observed by immunofluorescence analysis.

Next, we monitored the adenylate kinase (AK) activity that is released in the culture supernatant upon plasma membrane damage^22^. As expected, for both MR766 and PRVABC59 strains, we found a progressive increase of AK activity released in the supernatant as compared to uninfected controls (Fig. 6b).

Thus, both MR766 and, to lower extent, PRVABC59 strains establish productive infections that cause cytopathicity in myelinating DRG co-cultures.

### ZIKV infection induces a cellular stress response

**Figure 7:**
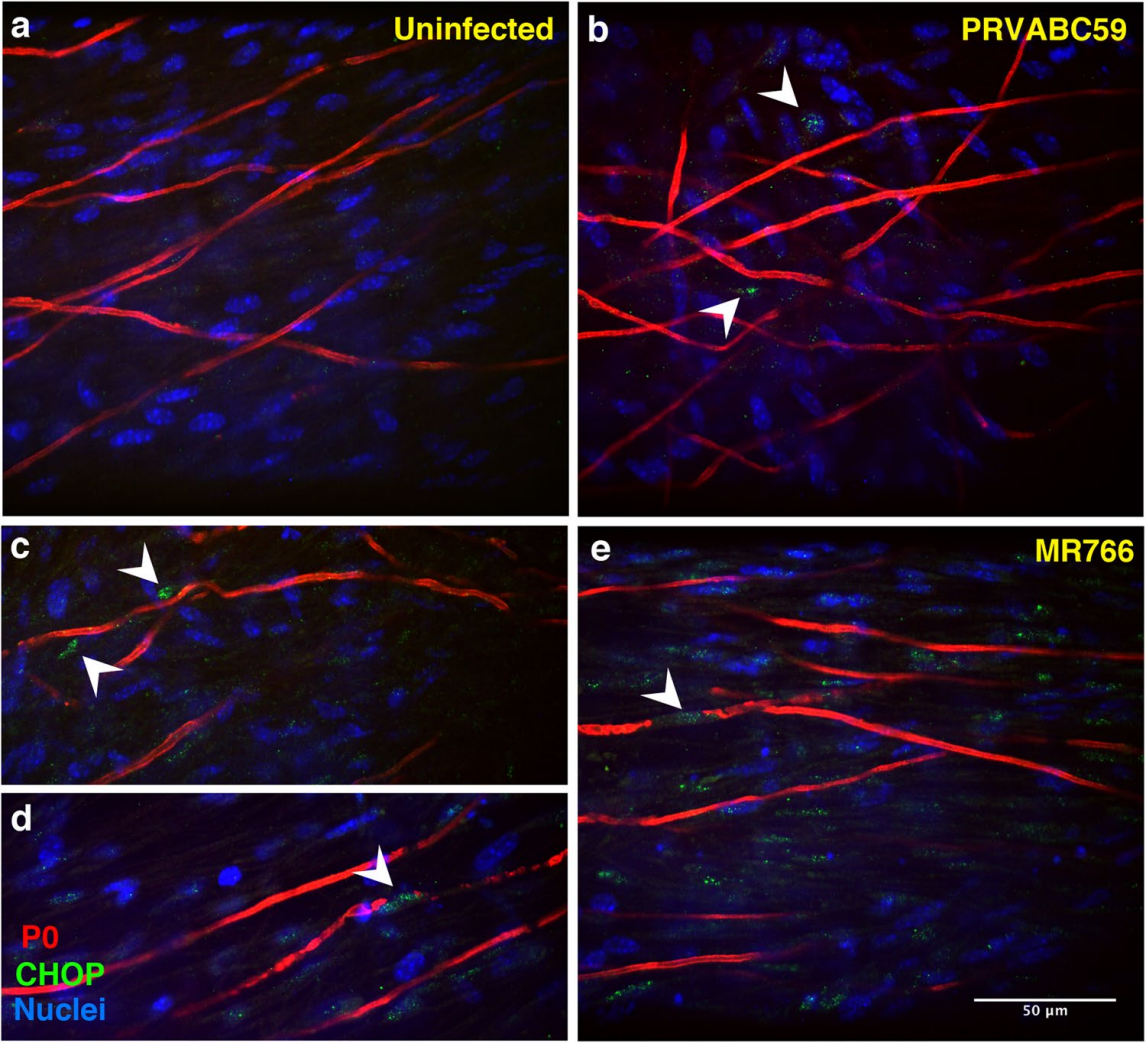
ZIKV infection activates the ER stress-mediated expression of CHOP in myelinating DRG co-cultures. Myelinating *Ifnar1*-KO DRG explants were infected with either PRVABC59 (**b**) or MR766 (**c**,**d**,**e**) strains after 2 weeks of myelination induction and compared to uninfected control (**a**). Staining with anti-CHOP (green) and anti-P0 (red) Abs was performed and Hoechst dye was used to stain the nuclei (blue). White arrowheads point at CHOP-positive nuclei detected at 3 dpi, index of activated ER stress response following ZIKV infection. Scale bar, 50 µm.

It has already been reported that ZIKV infection induces stress responses through the phosphorylation of the eukaryotic initiation factor 2-alpha (eIF2-alpha) leading to global translational arrest^23^. In addition, the phosphorylation of eIF2-alpha activates a number of transcription factors including activating transcription factor 4 (ATF4) that stimulates the expression of C/Ebp-Homologous Protein (CHOP)^24^. CHOP, a known pro-apoptotic transcription factor^25^, was previously shown to also modulate demyelination and cell death of SC in peripheral nerves in models of ER-stress related-neuropathies^26,27^. Since ZIKV proteins assemble in a replication complex in close association with ER proteins^28^, we tested whether ZIKV could cause ER stress in the infected DRG explants. Thus, we checked the expression of CHOP protein in the infected co-cultures by immunofluorescence. CHOP protein was not induced in cells of the uninfected control (Fig. 7a), whereas the nuclei of infected cells were positive after 3 dpi (Fig. 7b–e). Only a faint signal was detected upon infection with the contemporary PRVABC59 strain (Fig. 7b), whereas in the MR766-infected co-cultures, several PNS cells, many of which P0-positive myelinating SC, displayed signal of CHOP induction (Fig. 7c–e).

This evidence suggests that ZIKV infection can induce ER stress in our multicellular culture system by activating the P-eIF2-alpha/CHOP pathway, which may contribute to axon demyelination.

## Discussion

In the present study, we show that both the historical MR766 and the 2015 Puerto Rican PRVABC59 isolates establish a productive infection in DRG explants obtained from mice deficient in IFNAR1. Both neurons and SC were productively infected, although the MR766 infection was more efficient than that of the PRVABC59 isolate. Nevertheless, both viruses were cytopathic and caused myelin disruption. A stress response was triggered by ZIKV infection of SC cells as demonstrated by expression of CHOP in the nuclei of infected cells.

ZIKV can potentially reach the PNS through skin lesions and there are compelling evidences that the virus can cause peripheral nerve damage^18^. Despite the recent technology based on the re-programming of pluripotent stem cells to generate large quantities of human PNS cells^18,29^, to date there is no reliable protocol for the *in vitro* myelination of human induced Schwann cells (hiSCs) and neurons. Therefore, *ex vivo* myelination models have to be obtained from rodent DRG cultures^30^. In mice, however, the innate immune response to ZIKV and, in particular the type I interferon (IFN) response, significantly restricts virus infection and replication^21,31^. To overcome this restriction, many studies have exploited the *Ifnar1-*KO mice in which ZIKV infection of pregnant mice induces pathological changes of the progeny reminiscent of those observed in humans, regardless of whether the virus is inoculated intravenously, subcutanously or intravaginally^19,32–34^. In addition, ZIKV infection of adult *Ifnar1-*KO mice was found to reach the spinal cord and cause features of neurological disease, including hindlimb weakness and paralysis, before the infected mice succumbed to the infection^19,35^. In contrast with a recent study showing that DRG explants from *Ifnar1-*KO mice are poorly permissive to ZIKV infection^17^, we found productive infection of *Ifnar1*-KO DRG explants with both the historical MR766 and the recent 2015 Puerto Rican (PRVABC59) strains. Notably, the infection with the MR766 isolate was also highly detrimental, suggesting that mouse peripheral nerves are endowed with a strong innate IFN response, which either prevents or curtails ZIKV replication and pathogenicity. In line with these findings, immunocompetent myelinating DRG explants exposed for 10 days to comparable amounts of MR766 ZIKV did not show any sign of viral infection, replication and myelin loss (data not shown). Since both our study and that of Cumberworth *et al*.^17^ have been performed on DRG explants deriving from *Ifnar1-*KO mice, we exclude that the observed differences depend on a different origin of the DRG explants, although the two *Ifnar1-*KO lines are maintained on two distinct genetic backgrounds. Differently from Cumberworth *et al*.^17^, we measured the amount of the initial virus inoculum in terms of plaques forming units (PFU) rather than multiplicity of infection (moi), because the moi requires a precise determination of total target cell number that is difficult to obtain in a multicellular and multilayer culture system such as the DRG explants and we took advantage of two independent ZIKV strains rather than a single one^17^. In addition to a strong virus staining visualized by specific mAbs directed against either the viral E protein or the double-stranded RNA, we demonstrated that the infection was productive by quantifying the viral titers released in the supernatants collected every 3–4 days by plaque assay on Vero cells (lacking an IFN response^36^). Of note, however, is another recent study that showed productive ZIKV infection of sensory neurons and cytopathic infection of surrounding satellite glial cells in DRG cultures obtained from 6 week-old immunocompetent mice^37^. In contrast, as mentioned above, we did not detect any sign of viral infection and myelin loss in embryonic DRG obtained from immunocompetent mice but only from *Ifnar1*-KO mice. Overall, these results support a recent study demonstrating a potent intrinsic interferon-stimulated gene-dependent restriction, conserved across species, that protects embryonic stem cells from viral infections and wanes during the transition to terminally differentiated cells^38^.

Our results are in line with what published in a very recent work showing efficient ZIKV infection of DRG neurons in A129 mice and in both peripheral neurons and SC obtained from re-programming of human pluripotent stem cells^18^. At present, however, there is no established protocol to obtain the myelination of axons from human reprogrammed SC and to test whether SC infection with ZIKV alters myelin stability. In this regard, we observed that infection of *Ifnar1*-KO mouse myelinating SC causes a profound deterioration and fragmentation of the myelin sheath. It will be therefore important to determine whether ZIKV infection can disturb myelin stability also in humans.

ZIKV infection of peripheral neurons and SC also caused a potent cytopathic effect. The observed ZIKV-induced cell death in the DRG explants was mediated, at least in part, by induction of apoptosis, as shown by the activation of cl-CASP3. In this regard, other members of the *Flaviviridae* family as West Nile and Japanese encephalitis viruses have been reported to induce apoptosis in human SK-N-MC neuroblastoma and baby hamster kidney BHK21 cell lines, respectively^39,40^. This process might be due to persistent stress and unfolded protein response (UPR) activation in the ER, the cellular compartment in which viral protein synthesis, virion assembly and maturation take place^41^. Indeed very recently, ER stress and activation of the UPR was shown to contribute to ZIKV-associated microcephaly in humans and mouse models, by perturbing cortical neurogenesis and long-term neuronal survival^42^. Under prolonged virus-induced ER stress, pro-apoptotic pathways are activated by the induction of factors such as CHOP^39,40^. Indeed, CHOP was detected in the nucleus of cells in the infected DRG co-cultures 3 dpi. These results suggest that CHOP-mediated apoptosis might function to control ZIKV replication *in vitro* and this process may represent an important mechanism to counteract ZIKV replication and pathogenesis *in vivo*. Importantly, in a previous work, we demonstrated that CHOP induction underlies SC demyelination in a model of ER stress-related neuropathy^27^, suggesting that CHOP activation may also be involved in the extensive myelin disruption observed in the ZIKV infected cultures. In addition to apoptosis, however, the virus also causes cell necrosis in our *ex vivo* system, as demonstrated by increased levels of AK activity in the culture supernatant, released as a consequence of cell membrane damage. It remains to be determined whether the death mechanism involves either pyroptosis, leading to inflammasome-dependent activation of caspase 1 and release of interleukin-1β (IL-1β) and IL-18^43^, and/or programmed necrosis also defined as paraptosis-like death, as recently reported^44^.

ZIKV infection of myelinating mouse SC causes a strong disruption of the myelin membrane around the axons. This effect on *ex vivo* cultures is likely exaggerated as compared to the effects of ZIKV infection in patients. Indeed, the majority of ZIKV-associated GBS cases recovered after a few months suggesting that peripheral nerve SC can remyelinate axons and alleviate the symptoms^45^. However, it is important to emphasize that temporary loss of myelin might have long term effects that are still unrecognized. In this regard, a recent work has demonstrated that the disruption of SC in the auditory nerve of mice caused permanent hidden hearing loss even after myelin regenerated^46^ providing insights into potential long-term effects of acute demyelization occurring in GBS linked to ZIKV infection.

In conclusion, our study provides compelling evidence indicating that ZIKV can productively infect cells of the PNS causing cell death and myelin disruption, albeit in a system that is devoid of IFN-response. Nevertheless, myelinating DRG explants can provide relevant insights into ZIKV induced pathogenesis of the PNS.

## Methods

### Mouse breeding and genotyping

Mice lacking the type I interferon receptor (*Ifnar1*^−/−^ mice, inbred C57BL/6) have been previously described^47^. Mice were housed under specific pathogen-free conditions and used at 8–10 weeks of age. All experimental animal procedures were approved by the Institutional Animal Committee of the San Raffaele Scientific Institute. All methods were performed in accordance with the relevant guidelines and regulations.

### Myelinating Dorsal Root Ganglia (DRG) explants cultures

The DRG explants were obtained from E13.5 *Ifnar1*-KO embryos, seeded singly on rat collagen I-coated coverslips in 4-well plastic plates and maintained in culture at 37 °C, 5% CO_2_ as previously described^30^. Myelination was induced for 2–3 weeks by adding 50 μg/ml ascorbic acid (Sigma-Aldrich) to the culture medium. Culture medium was refreshed every two days. At least 7 independent coverslips per condition were used.

Infection of cultures with ZIKV

The viral strain MR766 was obtained from the European virus archive (EVAg) and the PRVABC59 strain was obtained from the CDC (GenBank Accession #KU501215). The viruses were subjected to three passages in Vero cells prior to generation of the viral stock used in this study. After 2 weeks of myelination induction, the culture medium was removed from the DRG explants and replaced with virus containing supernatant by adding 7 × 10^5^ PFU for the MR766 strain and 1.2 × 10^6^ PFU for the PRVABC59 strain. After 4 h, the supernatant was removed and fresh culture medium with 50 μg/ml ascorbic acid was added. The kinetics of virus replication were measured by plaque assay in supernatants harvested 1, 3, 6 and 10 dpi and maintained at −80 °C till testing.

Immunofluorescence and image capture

DRG explants were fixed 15 min in 4% paraformaldehyde solution (Sigma) in phosphate-buffered saline (PBS, Euroclone). The samples were permeabilized 5 min with ice cold methanol (>99.8%; Sigma) and then maintained 1 h in blocking solution containing 0.2% Triton X-100 (Sigma), 1% Bovine Serum Albumine (BSA, Sigma) and 10% normal goat serum (NGS, DAKO) in PBS. Primary antibodies were diluted in PBS containing 1% BSA, 0.1% Triton X-100 and incubated 1 h at room temperature. Secondary antibodies were diluted in 1% BSA in PBS and kept 45 min in dark condition; Hoechst dye was used to stain the nuclei. Slides were mounted on Vectashield (Vector Laboratories, H-1000) and stored at −20 °C until acquisition. Images were acquired with PerkinElmer UltraVIEW ERS microscope (40×-oil objective), taking advantage of Volocity 6.3 software and processed with both NIH ImageJ software and Adobe Photoshop CS4 (Adobe Systems, San Jose, CA).

### Antibodies

The following antibodies against Flavivirus E protein (1:200, mouse, Millipore, MAB10216), J2 double-stranded RNA (1:300, mouse, English and Scientific Consulting Kft, Hungary), Myelin Protein Zero (P0) (1:500, chicken, AVES labs, PZO), Neurofilament-H (1:200, rabbit, Millipore, AB1989), CHOP (1:600, mouse, 2895 S, Cell Signaling) and cl-CASP3 (1:300, rabbit, 9661, Cell Signaling) were used. Goat Anti-mouse FITC (1:200, SouthernBiotech, 1034–02), donkey anti-chicken TRITC (1:200, Jackson Immunoresearch, 703–025–155) and donkey anti-rabbit Cy5 (1:200, Jackson Immunoresearch, 711–175–152) were used as secondary antibodies.

### Plaque assay

Vero cells (1.2 × 10^6^) were seeded in 6-well culture plates. 24 h later, ten-fold dilutions of virus containing supernatants were prepared in culture medium supplemented with 1% heat-inactivated FBS and 1 ml of each dilution was added to the cells. The plates were incubated for 4 h at 37 °C. Unabsorbed virus was removed and 2 ml of culture medium supplemented with 1% methylcellulose (Sigma) were added to each well, followed by an incubation at 37 °C for 6 days. The methylcellulose overlay was removed and the cells were stained with 1% crystal violet in 70% methanol. Plaques were counted and viral titers were expressed as plaque-forming units per ml (PFU/ml).

### Cell death detection assay

10 μl samples of culture supernatant were transferred on a half black 96 well plate (Costar). To each well, 50 μl of the adenylate kinase detection reagent (ToxiLight^®^ BioAssay, Lonza) was added and the plate was incubated for 10 min at room temperature. Luminescence was measured in a Mithras LB940 Microplate Reader (Berthold Technologies). The results were expressed as relative luminescent unit (RLU).

### Statistical analysis

Prism GraphPad software v. 4.0 (www.graphpad.com) was used for all statistical analyses. Comparison among groups were performed using one-way ANOVA analysis of variance and the Bonferroni’s multiple comparison test.
